# Multicenter Case Registry Study of Lecanemab in Early Alzheimer's Disease in Northwest China

**DOI:** 10.1002/alz70861_108419

**Published:** 2025-12-23

**Authors:** Peijie Liu, Simeng CUI, Shan Wei, Ling Gao, Qiumin Qu, Jin Wang

**Affiliations:** ^1^ The First Affiliated Hospital of Xi'an Jiaotong University, Xi'an, Shaanxi China

## Abstract

**Background:**

Lecanemab (Leqembi®) is the first drug approved by FDA for the treatment of early AD (MCI due to AD or mild AD dementia) with confirmed brain amyloid pathology. The efficacy and safety of Lecanemab in China had not been reported.

**Method:**

This multi‐center case registry study was conducted at 30 hospitals in Northwest China. All AD patients treated with Lecanemab were enrolled. The efficacy of Lecanemab were evaluated using CDR‐SB, ADAS‐cog, ADCS‐ADL and MMSE every 3 months. ARIA were monitored via MRI‐T2‐FLAIR and MRI‐SWI. Blood samples were collected to evaluate the changes of plasma biomarkers (including Aβ_42,_ Aβ_40,_ Aβ_42/40_, *p* ‐tau181).

**Result:**

There were 118 participants received Lecanemab until April 20, 2025, all of whom were confirmed Aβ‐positive by CSF or PET scans. The mean age was 66.9 years (range 37‐85), with 79 (66.9%) female participants. Among the participants, 9 (7.6%) were homozygous for the ApoE ε4 allele, and 42 (35.6%) were heterozygous. At baseline, CDR‐SB: 4.3±2.3, MMSE: 18.1±6.1, ADAS‐cog: 18.4±10.4, ADCS‐ADL: 56.0±19.4. 63 participants completed the 3‐month follow‐up, 31 participants completed the 6‐month follow‐up, 9 participants completed the 9‐month follow‐up. The longest follow‐up duration was 42 weeks. There were no significant changes in cognitive scales among the participants completed 6‐month follow‐up. The change in CDR‐SB at 6 months compare to baseline was 0.46 (95%CI ‐1.07 to 1.99, *p* =0.524), MMSE: 5.33 (95%CI ‐1.00 to 2.07, *p* =0.469), ADAS‐cog: 1.67 (95%CI ‐1.78 to 5.13, *p* =0.315), and ADCS‐ADL: ‐1.29 (95%CI ‐6.56 to 3.99, *p* =0.573). The plasma Aβ_42/40_ was increased 0.016 (95%CI 0.003‐ 0.030, *p* =0.024), while plasma *p* ‐Tau 181 was decreased 0.77 (95%CI ‐1.37‐ ‐0.18, *p* =0.014) compare to baseline. **No ARIA has been reported**. Approximately 10% of patients reported treatment‐related adverse events primarily involved infusion‐related reactions, such as fever and chills, typically occurring within 24 hours post‐infusion, which were alleviated with symptomatic treatment.

**Conclusion:**

This real‐world study suggests that Lecanemab is well‐tolerated in clinical use in Northwest China, with manageable adverse effects and no severe adverse events such as ARIA reported to date. The neuropsychological scales keep stable during the 6‐month follow‐up, but plasma biomarkers showed significant improvement after treatment. A long time follow‐up and large sample are needed.

